# Frailty as a predictor of short-term adverse outcomes

**DOI:** 10.7717/peerj.1121

**Published:** 2015-07-30

**Authors:** Tiago Coelho, Constança Paúl, Robbert J.J. Gobbens, Lia Fernandes

**Affiliations:** 1 School of Allied Health Technologies, Polytechnic Institute of Porto, Porto, Portugal; 2 UNIFAI, ICBAS, University of Porto, Porto, Portugal; 3 Faculty of Health, Sports and Social Work, Inholland University of Applied Sciences, Amsterdam, The Netherlands; 4 Zonnehuisgroep Amstelland, Amstelveen, The Netherlands; 5 Department of Clinical Neurosciences and Mental Health, Faculty of Medicine, University of Porto, Porto, Portugal; 6 UNIFAI/CINTESIS Research Unit, Faculty of Medicine, University of Porto, Porto, Portugal

**Keywords:** Frailty, Frailty Phenotype, Groningen Frailty Indicator, Adverse outcomes, Tilburg Frailty Indicator

## Abstract

The objectives of this study were to compare how different frailty measures (Frailty Phenotype/FP, Groningen Frailty Indicator/GFI and Tilburg Frailty Indicator/TFI) predict short-term adverse outcomes. Secondarily, adopting a multidimensional approach to frailty (integral conceptual model–TFI), this study aims to compare how physical, psychological and social frailty predict the outcomes. A longitudinal study was carried out with 95 community-dwelling elderly. Participants were assessed at baseline for frailty, determinants of frailty, and adverse outcomes (healthcare utilization, quality of life, disability in basic and instrumental activities of daily living/ADL and IADL). Ten months later the outcomes were assessed again. Frailty was associated with specific healthcare utilization indicators: the FP with a greater utilization of informal care; GFI with an increased contact with healthcare professionals; and TFI with a higher amount of contacts with a general practitioner. After controlling for the effect of life-course determinants, comorbidity and adverse outcome at baseline, GFI predicted IADL disability and TFI predicted quality of life. The effect of the FP on the outcomes was not significant, when compared with the other measures. However, when comparing TFI’s domains, the physical domain was the most significant predictor of the outcomes, even explaining part of the variance of ADL disability. Frailty at baseline was associated with adverse outcomes at follow-up. However, the relationship of each frailty measure (FP, GFI and TFI) with the outcomes was different. In spite of the role of psychological frailty, TFI’s physical domain was the determinant factor for predicting disability and most of the quality of life.

## Introduction

As the number of elderly people increases worldwide, so does the prevalence of frailty (Clegg et al., 2013; Collard et al., 2012). This geriatric syndrome, particularly common in individuals older than 80 years, entails an increased risk of clinically significant adverse outcomes (Abellan van Kan et al., 2008; Morley et al., 2013; Rockwood & Mitnitski, 2007). Frail individuals are highly vulnerable, and minor stressful events can cause disability, institutionalization, hospitalization or even death (Abellan van Kan et al., 2008; Bergman et al., 2007; Clegg et al., 2013; Morley et al., 2013). Therefore, screening for frailty in the contexts of primary and community healthcare is fundamental to ensure the dignity and quality of life of older persons (Clegg et al., 2013; Morley et al., 2013).

There are different approaches regarding the conceptualization and operationalization of frailty (Bergman et al., 2007; Malmstrom, Miller & Morley, 2014; Morley et al., 2013; Rodriguez-Manas et al., 2013). The assessment based on the presence of the components that make up the Frailty Phenotype/FP (Fried et al., 2001) (unintentional weight loss, low physical activity, exhaustion, slow walking speed and weakness) has gained wide attention in the scientific community (Bouillon et al., 2013; Cesari et al., 2014; Rodriguez-Manas et al., 2013; Sternberg et al., 2011). This approach stems from a biological model, in which frailty is defined as an exclusively physical condition, caused by energy dysregulation and functional decline across multiple physiological systems (Fried et al., 2001; Fried, Walston & Ferrucci, 2009).

On the other hand, some authors argue that psychosocial factors can also increment vulnerability and lead to frailty (Abellan van Kan et al., 2008; Levers, Estabrooks & Ross Kerr, 2006; Markle-Reid & Browne, 2003; Sternberg et al., 2011). Furthermore, a biopsychosocial approach of a clinical syndrome such as frailty is more in line with the definition of health as physical, psychological and social well-being (Bergman et al., 2007; Gobbens et al., 2010a; Markle-Reid & Browne, 2003). Consequently, multidimensional measures, such as the Groningen Frailty Indicator/GFI (Schuurmans et al., 2004; Steverink et al., 2001) and the Tilburg Frailty Indicator/TFI (Gobbens et al., 2010e), have been developed as alternatives to the traditional physical operationalization. However, GFI and TFI present different pictures of frailty. In fact, while GFI includes functional performance/disability as components of the syndrome, in the integral conceptual model (Gobbens et al., 2010a; Gobbens et al., 2010b; Gobbens et al., 2010c; Gobbens et al., 2012b) (on which TFI is based) disability is regarded as a potential outcome of frailty. The clear distinction between frailty and disability is in consonance with a growing consensus in regard to frailty conceptualization (Hogan, MacKnight & Bergman, 2003; Sternberg et al., 2011; Walston et al., 2006).

Considering that there are different frailty measures, and that the prevention of frailty and its’ adverse effects is of the utmost importance from a social and public health perspective, research should focus on ascertaining which measures are most effective in detecting the syndrome and predicting outcomes in different populations (Bergman et al., 2007; Clegg et al., 2013; Pialoux, Goyard & Lesourd, 2012; Vermeulen et al., 2011). Therefore, the present study aims to compare how three well-known (Morley et al., 2013) tools (the FP, GFI and TFI) predict short-term adverse outcomes in a sample of community-dwelling elderly, particularly by analyzing whether the measures are associated with greater healthcare utilization and disability, and lower quality of life, in a 10-month follow-up. Additionally, assuming a multidimensional approach to frailty inherent to the integral conceptual model (Gobbens et al., 2010c), the secondary objective of this study is to examine which TFI’s domain (physical, psychological or social) is the most significant predictor of disability and quality of life. This model was chosen to assess frailty domains because it results from a recent and an exhaustive research (Gobbens et al., 2010a; Gobbens et al., 2010b; Gobbens et al., 2010c). In this approach, frailty is defined as a dynamic state affecting an individual who experiences physical, psychological and/or social losses, which is caused by a range of variables (decline in physiological reserves, diseases and/or life-course determinants), and which increases the risk of adverse outcomes (Gobbens et al., 2010c). Furthermore, studies have shown that TFI, which is based on the integral conceptual model, has a good predictive validity (Gobbens & Van Assen, 2012; Gobbens et al., 2012a; Gobbens et al., 2012b), and better psychometric properties than other multidimensional frailty questionnaires, such as the GFI and the Sherbrooke Postal Questionnaire (Metzelthin et al., 2010; Pialoux, Goyard & Lesourd, 2012).

## Materials & Methods

### Sample

From May to September 2013, a non-probabilistic sample of 252 community-dwelling elderly individuals (aged 65 years and over) was recruited from three northern Portuguese cities (Maia, Porto, V.N. Gaia). Exclusion criteria were severe cognitive impairment (screened with Mini Mental State Examination (Folstein, Folstein & McHugh, 1975; Guerreiro et al., 1994)) and inability to speak Portuguese, due to the self-report nature of most measures. The participants, users of local community institutions (e.g., social, recreation and day care centers), were interviewed by nine trained researchers. Among other measurements, the individuals were assessed for life-course determinants of frailty, comorbidity, frailty and adverse outcomes (disability, quality of life and healthcare utilization). In 2014, 10 months later, the 118 participants (47% of the total sample) who lived in the same geographical area (V.N. Gaia), were selected for a follow-up assessment, regarding the same adverse outcomes. From this group, only 95 individuals (38%) were included in the present study. After the first assessment, two participants died, five were admitted to a nursing home, two were hospitalized, one was ineligible due to severe cognitive impairment (severe dementia), nine could not be contacted, and four refused to participate. The follow-up interviews were conducted by three of the researchers that performed the first assessment. The study was approved by institutional review boards and written informed consent was obtained.

### Measures

Part A of TFI (Coelho et al., 2014; Gobbens et al., 2010e) was used to assess life-course determinants of frailty and comorbidity, while the FP (Fried et al., 2001), GFI (Duarte, 2013; Schuurmans et al., 2004; Steverink et al., 2001) and part B of TFI (Coelho et al., 2014; Gobbens et al., 2010e) were used to measure frailty. Disability in activities of daily living/ADL and in instrumental activities of daily living/IADL were measured with the Barthel Index (Araújo et al., 2007; Mahoney & Barthel, 1965) and with the Lawton and Brody Scale (Araújo et al., 2008; Lawton & Brody, 1969), respectively. Finally, quality of life was evaluated with EUROHIS-QOL-8 (Pereira et al., 2011; Schmidt, Muhlan & Power, 2006) and WHOQOL-OLD (Power et al., 2005; Vilar et al., 2010), whereas healthcare utilization was assessed with a set of questions previously used in other studies (Coelho et al., 2014; Gobbens & Van Assen, 2012; Gobbens et al., 2012a; Gobbens et al., 2012b; Gobbens et al., 2010e).

TFI (Gobbens et al., 2010e) consists of a self-report questionnaire divided into two parts. Part A (10 items) assesses determinants of frailty: sociodemographic characteristics (age, gender, marital status, ethnicity/nationality, education, income); life events in the last year (death of a loved one, serious illness, serious illness in a loved one, divorce or end of an important relationship, traffic accident, crime); assessment of how healthy the respondent’s lifestyle is; satisfaction with home living environment; and presence of two or more chronic diseases. Part B (15 items) measures physical frailty (physical health, unexplained weight loss, difficulty in walking, difficulty in maintaining balance, hearing problems, vision problems, lack of strength in hands, and physical tiredness), psychological frailty (cognition/memory, depression and anxiety symptoms, and coping), and social frailty (living alone, social relations and support). All items are rated dichotomously (0–1), and scores for each frailty domain and a total frailty score (0–15) are produced. Higher scores refer to higher frailty and, in the Portuguese version of TFI (Coelho et al., 2014), a cut-off of ≥6 can be used to classify elderly individuals as frail.

GFI (Schuurmans et al., 2004; Steverink et al., 2001) is also a questionnaire that aims to measure frailty in different domains: physical (difficulties in shopping, walking around outside, dressing and undressing, and going to the toilet, vision problems, and consumption of four or more medicines), cognition (complaints about memory), psychological (depressed mood and feelings of anxiety), and social (three items about emotional isolation). The Portuguese version (Duarte, 2013) has only 12 items, excluding three items from the original (physical fitness, hearing problems and weight loss). As for TFI, all items are rated dichotomously and scores refer to higher frailty (0–12). A cut-off point of ≥5 for frailty was considered (Duarte, 2013).

In regard to the assessment of the FP (Fried et al., 2001), unintentional weight loss was considered if the participant answered “yes” to TFI’s question 12 “Have you lost a lot of weight recently without wishing to do so?” Low physical activity and exhaustion were detected using two questions based on previous studies (Santos-Eggimann et al., 2009). Slow walking speed was detected if the participant took more than 20 s to complete the Timed Up and Go/TUG test (Podsiadlo & Richardson, 1991). Weakness was identified if the participant’s hand strength was below the cut-off determined by Fried et al. (2001), stratified by gender and BMI. In this regard, a GRIP-D Takei Hand Grip Dynamometer was used and a standardized approach for assessing hand strength was considered (Roberts et al., 2011). Frailty was identified if the participant had ≥3 components, and pre-frailty if one or two components were present.

The Barthel Index (Araújo et al., 2007; Mahoney & Barthel, 1965) (10 items) and the Lawton and Brody Scale (Araújo et al., 2007; Mahoney & Barthel, 1965) (8 items) are measures widely used to assess ADL and IADL disability, respectively, and, in both cases, lower scores refer to higher dependence. Regarding quality of life, the EUROHIS-QOL-8 (Pereira et al., 2011; Schmidt, Muhlan & Power, 2006) is an 8-item generic assessment instrument and the WHOQOL-OLD (Power et al., 2005) is a more extensive tool, of which the Portuguese version (Vilar et al., 2010) has 28 items distributed by 7 facets: sensory abilities; autonomy; past, present and future activities; social participation; death and dying; intimacy; family/family life. In both measures, higher scores indicate better quality of life. Finally, healthcare utilization was assessed in regard to the last year and with items regarding: contact with a general practitioner and with other healthcare professionals, hospitalization and different care support (professional personal, nursing, informal and in other healthcare or residential institutions). All answers were dichotomous (yes/no), except for contact with a general practitioner (0, 1–2, 3–4, 5–6 or ≥7 contacts). This item was later dichotomized for statistical analysis, and only ≥5 contacts were considered.

### Statistical analysis

Data is described using proportions, mean values and standard deviations, according to the nature of the variables. Independent samples *t*-test, chi-square test and Fisher’s exact test were used to compare the baseline characteristics (determinants of frailty, frailty, disability, quality of life, and healthcare utilization) of participants interviewed in the follow-up with those not reassessed. The comparison of the adverse outcomes at baseline and 10 months later was performed using paired samples *t*-test and McNemar test. Pearson’s correlation coefficient was calculated to examine the association between frailty measures, whereas Cohen’s kappa coefficient was analyzed to determine the agreement between the measures on classifying participants as frail. The association between healthcare utilization reported at follow-up with frailty at baseline was examined with chi-square test or Fisher’s exact test. Hierarchical regressions were conducted to analyze whether frailty at baseline predicted quality of life and disability in ADL and IADL at the 10-month reassessment, while controlling for the effect of life-course determinants, comorbidity and the same adverse outcome at baseline. Life-course determinants and comorbidity were included in the first step of the regression, and the same adverse outcome at baseline in the second. For these steps, the “enter” method was used. Variables that revealed low frequencies (<5%) were not included, neither was the life event serious illness because it overlaps comorbidity (Gobbens et al., 2012a; Gobbens et al., 2010d). Gender was classified as “1” for women and “0” for men. Marital status was classified as “1” for married/living with partner and “0” for unmarried, separated/divorced and widow/widower. Lifestyle was classified as “1” for healthy, “2” for not healthy, not unhealthy and “3” for unhealthy. The baseline scores of each frailty measure were included in the third step (step 3a). The “stepwise” method was used in order to ascertain which frailty measure improved prediction of the outcomes. The same procedure was used to examine which frailty domain improved the prediction of adverse outcomes at follow-up. In this case, while the first two steps were similar to the previous analysis, the third step (step 3b) consisted of including the scores obtained by TFI regarding physical, psychological and social frailty. Two-tailed tests were used throughout all analysis and a *p*-value <0.05 was considered statistically significant. All statistical analysis were conducted using IBM SPSS Statistics 22.0 (SPSS, Inc., Chicago, Ilinois, USA).

## Results

There were no statistically significant differences (regarding life-course determinants, comorbidity and frailty) between the individuals reassessed at follow-up and those who were not, except for gender and mean education years (Table 1).

**Table 1:** Baseline characteristics (life course-determinants, self-reported comorbidity and frailty) of the individuals that were reassessed at follow-up and those who weren’t.

	Participants		
	Assessed at follow-up (*n* = 95)	Not assessed at follow-up (*n* = 157)	
	*n* (%)	*n* (%)	*P*-value
**Life-course determinants and comorbidity**			
Age (years), mean ± SD	78.5 ± 6.2	79.6 ± 7.9	0.22^a^
65–74	24 (25.3)	44 (28.0)	
75–84	52 (54.7)	64 (40.8)	0.07^b^
≥85	19 (20.0)	49 (31.2)	
Gender (women)	64 (67.4)	127 (80.9)	<0.05^b^
Nationality (Portuguese)	95 (100)	156 (99.4)	1.00^c^
Marital status			
Married/living with partner	23 (24.2)	16 (16.6)	0.24^b^
Unmarried	9 (9.5)	15 (9.6)	
Separated/divorced	10 (10.5)	29 (18.5)	
Widow/widower	53 (55.8)	87 (55.4)	
Education (years), mean ± SD	3.6 ± 2.6	4.9 ± 4.1	<0.01^a^
0	15 (15.8)	21 (13.4)	
1–4	65 (68.4)	96 (61.1)	0.19^b^
≥5	15 (15.8)	40 (25.5)	
Monthly household income (euros)			
≤500	38 (40.0)	65 (41.4)	0.09^b^
501–750	28 (29.5)	22 (14.0)	
≥751	29 (30.5)	70 (44.6)	
Life events			
Death of a loved one	15 (15.8)	40 (25.5)	0.07^b^
Serious illness	25 (26.3)	31 (19.7)	0.22^b^
Serious illness in a loved one	29 (30.5)	42 (26.8)	0.52^b^
End of important relationship	3 (3.2)	5 (3.2)	1.00^c^
Traffic accident	1 (1.1)	0 (0.0)	0.38^c^
Crime	6 (6.3)	8 (5.1)	0.68^b^
Lifestyle self-assessment			
Healthy	59 (62.1)	78 (49.7)	0.13^b^
Not healthy, not unhealthy	30 (31.6)	62 (39.5)	
Unhealthy	6 (6.3)	17 (10.8)	
Satisfaction with living environment	78 (82.1)	121 (77.1)	0.34^b^
Self-reported comorbidity	49 (51.6)	85 (54.1)	0.69^b^
**Frailty**			
TFI total score (0–15), mean ± SD	5.6 ± 3.5	6.2 ± 3.4	0.18^a^
≥6 (Frailty)	46 (48.4)	92 (58.6)	0.12^b^
TFI physical domain score (0–8), mean ± SD	2.7 ± 2.2	3.0 ± 2.2	0.19^a^
TFI psychological domain score (0–4), mean ± SD	1.6 ± 1.1	1.8 ± 1.1	0.14^a^
TFI social domain score (0–3), mean ± SD	1.4 ± 1.1	1.4 ± 1.0	0.90^a^
GFI (0–12), mean ± SD	4.4 ± 2.4	4.7 ± 2.8	0.31^a^
≥5 (Frailty)	46 (48.4)	86 (54.8)	0.33^b^
FP, mean ± SD	1.8 ± 1.3	2.1 ± 1.4	0.09^a^
0 (Non-frailty/robustness)	15 (15.8)	24 (15.3)	
1–2 (Pre-frailty)	52 (54.7)	69 (43.9)	0.17^b^
≥3 (Frailty)	28 (29.5)	64 (40.8)	

**Notes.**

a Independent samples *t*-test.

b Chi-square test.

c Fisher’s exact test.

At baseline, the mean age of the participants that were reassessed (*n* = 95) was 78.5 years (±6.2). Most of them were women (67.4%), widowed (55.8%) and with a low level of education (0–4 years: 68.4%). The most common monthly household income was ≤500 euros (40.0%) and the most shared life event was serious illness in a loved one (30.5%). Most rated their lifestyle as healthy (62.1%) and were satisfied with their living environment (82.1%). Comorbidity was reported by 51.6% of the participants. The mean TFI total score was 5.6 (±3.5) and the mean GFI total score was 4.4 (±2.4). The prevalence of frailty in this group ranged from 29.5% (detected with the FP) to 48.4% (measured with GFI and TFI) (Table 1).

In regard to the association between frailty measures, TFI and GFI were strongly correlated (*r* = 0.77, *p* < 0.001). On the other hand, the FP was only moderately associated with the remaining measures (TFI: *r* = 0.49, *p* < 0.001; GFI: *r* = 0.51, *p* < 0.001). Likewise, there was an agreement of 85.3% between TFI and GFI (*k* = 0.70, *p* < 0.001, 95% CI [0.56–0.85]), but only of 60% between the FP and TFI (*k* = 0.19, *p* < 0.05, 95% CI [0–0.39]), and of 64.3% between the FP and GFI (*k* = 0.27, *p* < 0.01, 95% CI [0.08–0.47]).

Regarding the comparison of the outcomes at baseline and at follow-up, only IADL disability, the autonomy facet of quality of life and three indicators of healthcare utilization (contact with healthcare professionals, hospitalization and nursing care) showed significant differences (Table 2).

**Table 2:** Adverse outcomes at baseline and at follow-up.

Outcome	Baseline		Follow-up		Δ (difference)		*P*-value^a^
	M	SD	M	SD	M	SD	
**Quality of life**							
EUROHIS-QOL-8	27.7	4.8	27.4	4.7	−0.4	4.1	0.40
WHOQOL-OLD	99.2	16.1	99.2	15.3	0.0	9.4	1.00
Sensory abilities	15.6	4.0	16.1	3.6	0.5	3.3	0.12
Autonomy	14.1	3.2	14.9	3.0	0.8	2.9	<0.05
Past, present and future activities	13.5	3.9	13.2	2.7	−0.2	2.7	0.42
Social participation	15.0	2.9	14.8	2.8	−0.3	2.5	0.27
Death and dying	12.6	4.5	13.1	4.1	0.4	3.9	0.31
Intimacy	13.4	4.0	12.6	4.1	−0.8	4.2	0.08
Family/family life	15.0	4.0	14.5	3.5	−0.4	2.6	0.10
**ADL disability**							
Barthel Index	19.2	1.3	19.0	1.4	−0.2	1.4	0.23
**IADL disability**							
Lawton and Brody scale	18.0	5.2	15.1	6.4	−2.9	3.7	<0.001

	*n*	**%**	*n*	**%**	**%**	*P*-value^b^
**Healthcare utilization**						
Contact with general practitioner (≥5)	17	17.9	11	11.6	−6.3	0.21
Contact with healthcare professionals	69	72.6	55	57.9	−14.7	<0.05
Hospitalization	24	25.3	11	11.6	−13.6	<0.01
Professional personal care	9	9.5	6	6.3	−3.2	0.55
Nursing care	29	30.5	10	10.5	−20.0	<0.001
Informal care	19	20.0	19	20.0	0.0	1.00
Other healthcare/residential institutions	10	10.5	4	4.2	−6.3	0.11

**Notes.**

a Paired samples *t*-test.

b McNemar test.

There was a significant relationship between being classified as frail at baseline and indicators of healthcare utilization at follow-up (Table 3). Each measure of frailty was associated with one specific indicator: the FP with a greater utilization of informal care; GFI with increased contact with healthcare professionals; and TFI with a greater contact with a general practitioner.

**Table 3:** Frailty at baseline and healthcare utilization at follow-up.

Healthcare utilization at follow-up		FP		*P*-value	GFI		*P*-value*	TFI		*P*-value
		Non-frail	Frail		Non-frail	Frail		Non-frail	Frail	
		*n* (%)	*n* (%)		*n* (%)	*n* (%)		*n* (%)	*n* (%)	
Contact with general practitioner (≥5)	Yes	8 (72.7)	3 (27.3)	1.00^b^	4 (36.4)	7 (63.6)	0.28^a^	2 (18.2)	9 (81.8)	<0.05^a^
	No	59 (70.2)	25 (29.8)		45 (53.6)	39 (46.4)		47 (56.0)	37 (44.0)	
Contact with healthcare professionals	Yes	39 (70.9)	16 (29.1)	0.92^a^	22 (40.0)	33 (60.0)	<0.01^a^	24 (43.6)	31 (56.4)	0.07^a^
	No	28 (70.0)	12 (30.0)		27 (67.5)	13 (32.5)		25 (62.5)	15 (37.5)	
Hospitalization	Yes	6 (54.5)	5 (45.5)	0.29^b^	3 (27.3)	8 (72.7)	0.08^a^	3 (27.3)	8 (72.7)	0.09^a^
	No	61 (72.6)	23 (27.4)		46 (54.8)	38 (45.2)		46 (54.8)	38 (45.2)	
Professional personal care	Yes	3 (50.0)	3 (50.0)	0.36^b^	2 (33.3)	4 (66.7)	0.43^b^	2 (33.3)	4 (66.7)	0.36^b^
	No	64 (71.9)	25 (28.1)		47 (52.8)	42 (47.2)		47 (52.8)	42 (47.2)	
Nursing care	Yes	6 (60.0)	4 (40.0)	0.47^b^	6 (60.0)	4 (40.0)	0.74^b^	4 (40.0)	6 (60.0)	0.52^b^
	No	61 (71.8)	24 (28.2)		43 (50.6)	42 (49.4)		45 (52.9)	40 (47.1)	
Informal care	Yes	7 (36.8)	12 (63.2)	<0.001^a^	7 (36.8)	12 (63.2)	0.15^a^	7 (36.8)	12 (63.2)	0.15^a^
	No	60 (78.9)	16 (21.1)		42 (55.3)	34 (44.7)		42 (55.3)	34 (44.7)	
Other healthcare/residential institutions	Yes	2 (50.0)	2 (50.0)	0.58^b^	2 (50.0)	2 (50.0)	1.00^b^	1 (25.0)	3 (75.0)	0.35^b^
	No	65 (71.4)	26 (28.6)		47 (51.6)	44 (48.4)		48 (52.7)	43 (47.3)	

**Notes.**

a Chi-square test.

b Fisher’s exact test.

Regarding the regression analysis (Table 4) ethnicity/nationality was not included because all participants were Portuguese. Life events: divorce or end of an important relationship and traffic accidents, were also excluded because of their low frequency. The remaining life events were grouped (“1” for death and/or serious illness in a loved one, and “0” for absence of these life events).

**Table 4:** Prediction of outcomes (disability and quality of life) in a 10-month follow-up by life-course determinants and comorbidity (step 1), by the same outcome at baseline (step 2), by the Frailty Phenotype/FP, Groningen Frailty Indicator/GFI and the Tilburg Frailty Indicator/TFI.

Predictors	Barthel Index	Lawton and Brody scale	EUROHIS-QOL-8	WHOQOL-OLD							
				Total	Sensory abilities	Autonomy	Past, present and future activities	Social participation	Death and dying	Intimacy	Family/ family life
**Step 1** (enter)											
Age (years)	−0.06^*^	−0.26^*^	0.12	0.17	−0.05	0.00	0.08	0.04	−0.12	0.07	0.16^*^
Gender (women vs men) ^a^	−0.41	−0.67	0.68	0.49	0.25	−0.11	0.45	0.22	−0.56	−0.68	0.92
Education (years)	−0.05	0.05	0.25	0.15	0.16	0.16	−0.02	−0.12	−0.09	−0.05	0.10
Marital status (married vs unmarried) ^b^	−0.21	−2.65	−0.01	1.64	0.08	−1.01	0.09	0.03	−1.11	2.85^*^	0.76
Household income	0.12	−0.53	−0.33	0.29	0.27	0.06	0.16	0.05	0.04	−0.19	−0.09
Life events (yes vs no)^c^	−0.13	0.00	−0.84	−1.34	−0.20	−0.68	−0.38	−0.34	−0.54	−0.09	0.89
Lifestyle self-assessment^d^	−0.08	−1.01	−2.15^**^	−9.24^***^	−0.88	−1.99^***^	−1.01^*^	−1.21^*^	−1.79^*^	−0.74	−1.64^**^
Satisfaction living environment (yes vs. no)	−0.33	−2.35	1.52	8.80^*^	1.36	0.27	1.60^*^	1.01	1.00	2.47^*^	1.04
Self-reported comorbidity (yes vs. no)	−0.18	1.45	−2.33^*^	−0.03	−0.13	0.60	0.06	0.19	−1.35	0.51	0.08
Δ*R*^2^ (%)	9.8	17.0	24.9^**^	22.0^**^	8.5	17.3	18.7^*^	13.7	15.7	18.2^*^	21.5^*^
**Step 2** (enter)											
Outcome at baseline	0.51^***^	1.00^***^	0.53^***^	0.82^***^	0.58^***^	0.51^***^	0.43^***^	0.62^***^	0.49^***^	0.41^***^	0.70^***^
Δ*R*^2^	19.5^***^	55.0^***^	21.4^***^	47.2^***^	33.9^***^	22.8^***^	17.1^***^	28.6^***^	22.7^***^	12.5^***^	41.1^***^
**Step 3a** (stepwise)											
FP	−	−	−	−	−	−	−	−	−	−	−
GFI	−	−0.39^*^	−	−	−	−	−0.39^***^	−0.39^***^	−	−	−
TFI	−	−	−0.42^**^	−1.09^**^	−0.29^*^	−	−	−	−	−0.32^*^	−0.27^**^
Δ*R*^2^(%)	−	1.4^*^	4.4^**^	2.4^**^	3.3^*^	−	8.6^***^	7.2^***^	−	4.4^*^	4.4^**^
*R*^2^ (%) total	29.3^***^	73.4^***^	50.7^***^	71.5^***^	45.7^***^	40.1^***^	44.4^***^	49.5^***^	38.4^***^	35.2^***^	66.9^***^
**Step 3b** (stepwise)											
Physical frailty	−0.15^*^	−0.55^**^	−0.60^**^	−1.65^**^	−0.49	−	−	−0.38^**^	−0.39^*^	−	−0.32^*^
Psychological frailty	−	−	−	−	−	−	−0.79^**^	−	−	−0.87^*^	−
Social frailty	−	−	−	−	−	−	−	−	−	−	−
Δ*R*^2^ (%)	3.3^*^	2.2^**^	4.7^**^	2.9^**^	4.5^**^	−	7.2^**^	5.6^**^	3.0^*^	4.0^*^	2.8^*^
*R*^2^(%) total	32.6^***^	74.2^***^	51.0^***^	72.1^***^	46.9^***^	40.1^***^	42.9^***^	47.8^***^	41.4^***^	34.8^***^	65.4^***^

**Notes.**

a Gender (“1” for women and “0” for men).

b Marital status (“1” for married/living with partner and “0” for unmarried, separated/divorced and widow/widower).

c Life events (“1” for death and/or serious illness in a loved one, and “0” for absence of these life events).

d Lifestyle (“1” for healthy, “2” for not healthy, not unhealthy and “3” for unhealthy).

Regression coefficients (b) are displayed.

* *p* < 0.05.

** *p* < 0.01.

*** *p* < 0.001.

Frailty measures explained from 29.3% to 73.4% of the variances of the disability and quality of life scores. After controlling for the effect of life-course determinants, comorbidity and the same adverse outcome at baseline, the TFI was most often selected as the measure that better predicted the outcomes. After the TFI was inserted in the regression models, an additional 4.4% of quality of life variance (measured by EUROHIS-QOL-8) and 2.4% (measured by WHOQOL-OLD), was explained. The TFI also predicted three quality of life (WHOQO-OLD) facets: sensory abilities (3.3%), intimacy (4.4%) and family/family life (4.4%). On the other hand, GFI was the measure that most significantly increased the prediction of IADL disability (1.4%) and two quality of life facets: past, present and future activities (8.6%) and social participation (7.2%). The effect of the FP on the outcomes was not significant, when compared to the other measures. Concomitantly, neither of the frailty measures predicted ADL disability and quality of life facets: autonomy, and death and dying. In summary, an increment in frailty was associated with a decrease in quality of life and an increase in disability.

When comparing TFI’s frailty domains, physical frailty contributed to the prediction of most of the adverse outcomes: ADL disability (3.3%), IADL disability (2.2%), global quality of life (EUROHIS-QOL-8: 4.7%; WHOQOL-OLD: 2.9%) and quality of life facets: sensory abilities (4.5%), social participation (5.6%), death and dying (3.0%) and family/family life (2.8%). On the other hand, TFI’s psychological domain predicted past, present and future activities (7.2%) and intimacy (4.0%), whereas the effect of social frailty was not significant.

## Discussion

In general, frailty at baseline was associated with the adverse outcomes at follow-up. The TFI predicted global quality of life, the GFI predicted disability, while the FP was not relevant after controlling for comorbidity and the remaining frailty measures. On the other hand, when comparing TFI’s frailty domains, physical frailty was the most significant predictor of the outcomes, even explaining part of the variance of ADL disability.

As in previous studies (Malmstrom, Miller & Morley, 2014; Metzelthin et al., 2010; Theou et al., 2013), each frailty measure classified a different group of individuals as frail. This is due to the fact that each measure operationalizes frailty through a different set of components. Consequently, as expected (Theou et al., 2013), the agreement between biopsychosocial measures of frailty (TFI and GFI) was higher than between the FP and these measures. Considering this variability, the choice of frailty measure should take into account its feasibility and predictive ability.

A successful operationalization of frailty, among other factors, implies that it is easy to use in a busy clinical setting, and allows the prediction of adverse outcomes (Rockwood, 2005). From this standpoint, the assessment of the FP is immediately at disadvantage, as it seems less practical than administering GFI or TFI, due to requiring the measurement of grip strength and gait speed (Cesari et al., 2014; Malmstrom, Miller & Morley, 2014). Furthermore, in the present study, despite being associated with increased utilization of informal care, the FP’s contribution to the prediction of disability and quality of life was inferior when compared with the other measures. This does not mean that the FP would not be able to predict outcomes in different time periods (i.e., medium and-long term), or other adverse outcomes. In fact, several studies (Fried et al., 2001; Kulmala, Nykanen & Hartikainen, 2014; Ravindrarajah et al., 2013; Theou et al., 2013; Woo, Leung & Morley, 2012) have shown that it predicts outcomes such as falls, disability, hospitalization and mortality, in different time frames. The present study shows that multidimensional measures of frailty assessment were better predictors of the selected outcomes in a 10-month follow-up than an exclusively physical one. This may be related not only to the components of the GFI and the TFI, but also to the amplitude of their scores, since measures with continuous scores seem to discriminate better between frail and non-frail individuals (Cesari et al., 2014; Clegg et al., 2013; Kulminski et al., 2008).

Indeed, besides being associated with increased contact with healthcare professionals, the GFI explained the variance of IADL disability. However, the fact that its contribution to the prediction of disability is greater than the other measures (which is consistent with another study (Daniels et al., 2012)) may simply be justified by the inclusion of four disability related questions in the GFI itself. Nevertheless, there is some evidence (Malmstrom, Miller & Morley, 2014) that by including the assessment of comorbidity there is a relevant increase in the prediction of disability. Moreover the GFI comprises a question about the consumption of four or more medicines, which is directly related to the presence of multiple diseases. On the other hand, the TFI was associated with a greater contact with a general practitioner and independently predicted global quality of life, which is consistent with previous studies (Gobbens, Luijkx & Van Assen, 2012; Gobbens & Van Assen, 2012; Gobbens & Van Assen, 2014; Gobbens et al., 2012a; Gobbens et al., 2012b). The prediction of an intricate concept such as quality of life emphasizes the relevance of a holistic definition of frailty and of the TFI’s components (Gobbens, Luijkx & Van Assen, 2012; Gobbens & Van Assen, 2014).

In regard to the comparison of TFI’s frailty domains, similarly to prior research (Gobbens, Luijkx & Van Assen, 2012; Gobbens & Van Assen, 2012; Gobbens & Van Assen, 2014; Gobbens et al., 2012a; Gobbens et al., 2012b), the physical domain provided the most important contribution for the explanation of the variance of the adverse outcomes. Nevertheless, while it has been previously observed (Gobbens, Luijkx & Van Assen, 2012; Gobbens & Van Assen, 2012; Gobbens & Van Assen, 2014; Gobbens et al., 2012b) that the TFI’s psychological and social domains also predicted disability and quality of life, in the present study, only the contribution of psychological frailty was significant, as it was independently associated with two quality of life facets. These results highlight the relevance of physical factors, but also the importance of including at least psychological components in the definition of frailty.

On the other hand, it should be emphasized that the TFI’s physical domain explained ADL disability and the death and dying facet of quality of life, whereas other global frailty measures were unable to do so. First, this may suggest that the components of the TFI’s physical domain circumscribed physical frailty more precisely than the FP’s components (i.e., the components of the FP might have been insufficient to predict the outcomes). Second, it may indicate that the TFI’s psychological and/or social domains include items that were detrimental to the prediction of these specific outcomes. Nonetheless, the fact that, in some cases, the contribution of the TFI’s psychological domain was more important than the physical one, and that in previous studies (Gobbens, Luijkx & Van Assen, 2012; Gobbens & Van Assen, 2012; Gobbens & Van Assen, 2014; Gobbens et al., 2012b) the social domain explained some of the outcomes’ variance, justify TFI’s multidimensional structure.

A comprehensive approach of frailty, inherent to the integral conceptual model on which TFI is based, is critical avoid the fragmentation of care for older adults (Gobbens et al., 2010c). Indeed, screening for an exclusively physical condition would leave out those who are highly vulnerable due to the lack of psychological and/or social resources. This should be taken into account when planning community healthcare services.

The main strengths of the present study are related to its longitudinal design and to the fact that the prediction of disability and quality of life was examined after controlling for the effect of life-course determinants, comorbidity and the same adverse outcome at baseline. It is also the first study to examine how TFI’s domains predict adverse outcomes in elderly individuals from a southern European country. Nonetheless, some limitations should be noted. First, the non-probabilistic sampling method could limit the generalization of results. Second, the relatively small sample size limited the analysis of the prediction of dichotomous variables such as the healthcare utilization indicators, after adjusting for baseline characteristics, as it was done for scale scores (disability and quality of life). Third, outcomes were only assessed through self-report, which in part might explain why they were mainly associated with the exclusively self-report measures (to the detriment of the FP, which included objective measurements). Finally, the selected operationalization of the FP was different from other studies (Fried et al., 2001; Malmstrom, Miller & Morley, 2014; Santos-Eggimann et al., 2009), which limits the comparability of the results. Furthermore, the hand strength cut-off points used were based on the original study regarding the FP.

Several directions for future research can be suggested. Studies should focus on examining the prediction of outcomes in different time frames (medium and long-term). Other outcomes such as falls, institutionalization and mortality should also be analyzed. Likewise, the association between physical, psychological and social frailty components and different adverse outcomes should be better examined in order to improve the understanding of the multidimensional nature of frailty.

## Conclusions

The choice of a frailty measure should take into account its ability to predict specific adverse outcomes. In the present study, considering a time interval of 10 months, GFI predicted an increase in IADL disability, and TFI predicted a decline in quality of life, in a sample of community-dwelling elderly individuals. In turn, this study demonstrated that although there are benefits in using multidimensional frailty measures, the physical components of frailty seem to have greater importance for the prediction of adverse outcomes.

## Additional Information and Declarations

### Data Deposition

The following information was supplied regarding the deposition of related data: The raw data cannot be provided because it refers to clinical observations of our elderly participants. This is fundamental to ensure the confidentiality of data and anonymity of the participants.
